# Predictive value and dynamic risk stratification of high sensitive basal or stimulated thyroglobulin assay in a long-term thyroid carcinoma cohort

**DOI:** 10.1007/s12020-023-03320-y

**Published:** 2023-02-23

**Authors:** Pablo Fernández-Velasco, Gonzalo Díaz-Soto, Paloma Pérez López, Beatriz Torres Torres, Daniel de Luis

**Affiliations:** 1 Endocrinology and Nutrition Department, Hospital Clínico Universitario de Valladolid, Valladolid, Spain; 2 Centro de Investigación de Endocrinología y Nutrición Clinica (CIENC), Facultad de Medicina. Universidad de Valladolid, Valladolid, Spain

**Keywords:** Dynamic Risk Stratification, Thyroid cancer, thyroglobulin, excellent response

## Abstract

**Purpose:**

To evaluate the predictive value of the rhTSH thyroglobulin stimulation test (rhTSH-Tg) compared to basal high-sensitive thyroglobulin (hs-Tg) under TSH suppressive therapy at 12 months after the completion of initial treatment to predict the long-term response and Dynamic Risk Stratification (DRS) at the last follow-up visit in a long-term DTC cohort.

**Methods:**

Prospective study in 114 DTC patients (77.2% women, mean age 46.4 ± 14.1 years old, median/IQR evolution 6.7[3.1–8.0] years) from 2013 to 2020 undergoing total thyroidectomy and radioiodine ablation in whom hs-Tg and rhTSH-Tg was performed 12 months after completing initial treatment. Pearson correlation, receiving operating characteristics (ROC) and DRS at initial and last follow-up visit were analyzed.

**Results:**

hs-Tg and rhTSH-Tg show a strong positive linear correlation (*r* = 0.864, *p* < 0.001). The diagnostic performance of initial hs-Tg and rhTSH-Tg levels were evaluated via ROC-AUC as a predictor of excellent response (ER) in the last follow-up visit. Hs-Tg showed a better AUC (0.969, 95%CI = 0.941–0.997) than rhTSH-Tg (0.944, 95%IC = 0.905–0.984; *p* < 0.001). The hs-Tg and rhTSH-Tg cutoff point of highest sensitivity (S) and specificity (E) was 0.110 and 0.815 ng/dl, respectively. Hs-Tg showed a higher diagnostic accuracy than rhTSH-Tg (S = 100% vs 96.8%, E = 84.3% vs 84.3%, NPV = 100% vs 98.6%, PPV = 70.5% vs 69.7%; *p* < 0.05). The DRS based on initial hs-Tg showed better ability to predict ER (93.3% vs 86.7%) and biochemical incomplete response (53.3%vs13.3%) in the last follow-up visit compared to rhTSH-Tg.

**Conclusions:**

Both initial hs-Th and rhTSH-Tg were good predictors of long-term ER. In patients with hs-Tg, the rhTSH-test did not provide relevant prognosis information. An ER after initial treatment was associated with a very high NPV at subsequent follow-up.

## Introduction

Even though differentiated thyroid carcinoma (DTC) is the most frequent malignant endocrine neoplasia, the prevalence in the general population is less than 1%, mostly affecting middle aged women, but generally showing an excellent prognosis [1, 2]. In this context, international clinical guidelines have updated their protocols for the diagnosis, treatment and follow-up of thyroid nodules and DTC towards less aggressive methods of control (2).

Long-term management of DTC after total thyroidectomy has involved ablative treatment with [131]I and suppression therapy of TSH levels with levothyroxine at supraphysiologic doses in virtually all patients. In addition, a long-term clinical follow-up program was considered mandatory to detect persistent or recurrent disease by performing total body [131]I scans, cervical ultrasound and the assessment of thyroglobulin (Tg) and anti-thyroglobulin antibody (AbTg) levels every 6–12 months [2]. However, nowadays, the greater sensitivity of Tg assays and cervical ultrasound imaging has reduced the use of the remaining techniques to special situations (presence of positive AbTg, diagnostic doubts, among others) [2, 3].

Second, survival prediction models in DTC based exclusively on initial diagnostic factors have not shown sufficient accuracy for long-term assessment due to their static characteristics [4]. Static risk stratification systems predict disease-specific risk of recurrence and mortality based solely on clinical, pathologic, and radiologic features at initial management. The development of dynamic risk stratification (DRS) models that take into account biochemical and radiological changes during long-term follow-up allows the response to treatment to be reassessed at any time, as well as identifying those patients who may require a closer follow up [5]. Therefore, the DRS assesses the biological/histological features of DTC, the treatment response at any time, and the minimum follow-up required [6].

Although, Tg (with negative AbTg) is the essential marker of persistence/recurrence in DTC [7], due to the low analytical functional sensitivity of first generation Tg assays, their measurements required the stimulation of basal Tg levels under TSH suppressive therapy by prolonged thyroid hormone withdrawal (THW) or through the administration of recombinant human TSH (rhTSH). In fact, the 2015 American Thyroid Association (ATA) Clinical Guideline recommended that all patients should undergo Tg stimulation tests 6–12 months after [131]I ablation therapy [2]. However, both stimulation procedures are uncomfortable for the patient, time consuming and require strict protocols with a high cost of human and economic resources.

Recently, high-sensitivity Tg assays (hs-Tg) have been developed with a functional sensitivity (FS) tenfold higher than Tg conventional assays (0.1 vs 1.0 ng/mL, respectively) [8]. The use of hs-Tg in DTC may be associated with some advantages, such as avoiding the use of stimulated Tg tests [8] or the earlier detection of recurrence [9]. However, this additional sensitivity may present some disadvantages such as the detection of low levels of serum Tg with no clear clinical implication, which may normalize spontaneously over time. In addition, the DRS can be classified according to either basal or stimulated Tg levels; thus, differences in staging can be observed depending on the specific Tg assessed. The clinical implications of these divergences are currently unknown [2].

The aim of the present study was to evaluate the ability of rhTSH-Tg stimulation test compared to basal hs-Tg on levothyroxine treatment at 12 months after completion of the initial treatment to predict the long-term response and DRS at the last follow-up visit in a long-term DTC cohort.

## Methods

A prospective longitudinal cohort study was carried out in all patients with DTC in follow-up on the Thyroid Nodule and Cancer Clinic on the Endocrinology and Nutrition Department at a Tertiary Hospital. All DTC diagnosed from January 2013 to December 2020 with a rhTSH stimulation tests performed 12 months after completing initial treatment (thyroidectomy and [131]I ablation) were included.

The following clinical data were collected: age at diagnosis, year of thyroid cancer diagnosis, duration of clinical course in months from diagnosis to the last visit, type and characteristics of the thyroid cancer diagnosed: size in centimeters, multifocality, capsular and vascular invasion and TNM staging. In addition, the following were recorded: risk of recurrence following diagnosis (excluding molecular markers) and DRS at 12 months after the completion of initial treatment and at the last monitoring visit during 2022, according to the ATA 2015 guideline [2], in those patients who underwent surgery with total thyroidectomy and ablation with radioactive iodine.

Histological classification was performed according to the WHO classification [10] in 2 main categories: papillary carcinoma and follicular carcinoma. The latter includes minimally and widely invasive variants, the clear cell variant and Hürthle cell carcinomas (or oncocytic variant). Histological variants of papillary carcinoma were grouped into 3 categories: classic papillary carcinomas, follicular variant papillary carcinomas and the most aggressive histologic variants (diffuse sclerosing, solid-trabecular, tall cell and columnar cell subtypes). Tumors were classified at diagnosis according to the 8th edition of the American Joint Committee of Cancer TNM classification [11].

Laboratory parameters for DTC monitoring were analyzed by an electrochemiluminescence immunoassay with the same methodology throughout the entire follow-up (ECLIA) (Roche Diagnostics, Geneva, Switzerland). In particular, second-generation plasma Tg, AbTg and circulating TSH levels 12 months after completing initial treatment and at the last follow-up visit on levothyroxine treatment.

Serum basal Tg on levothyroxine treatment according to TSH targets recommended by 2015 ATA Guidelines [2, 25] and stimulated Tg were determined 12 months after completing initial treatment (total thyroidectomy and ablation with [131]I) using the second-generation ultrasensitive immunoassay Elecsys® on Cobas® e801 according to the manufacturer’s instructions (Roche Diagnostics, Geneva, Switzerland). As a positive serum Tg result, we adopted the FS of the test (0.1 μg/L), understood as the minimum concentration of Tg that can be evaluated with an inter-test imprecision (coefficient of variation <20%), with Tg values <0.1 ng/mL being considered undetectable.

The rhTSH stimulation test consisted of the administration of an intramuscular injection of rhTSH (0.9 mg im, Thyrogen®; Genzyme Cambrige, USA) for 2 consecutive days. Serum samples for the measurement of TSH, Tg and AgTb were collected on the first day of the stimulation test immediately before the rhTSH injection and 3 days after the second rhTSH injection. In addition, to verify adequate stimulation with rhTSH, TSH levels were determined 72 hours after the first rhTSH injection.

All patients signed an informed consent for their inclusion before participating in the study. The protocol was approved by the Clinical Research Ethics Committee of the Hospital Center (PI 21-2404) and the study was conducted in accordance with the Declaration of Helsinki.

### Statistical analysis

The quantitative data were expressed as mean and standard deviation (SD), except in those cases with a non-normal distribution that were expressed as median and interquartile range (IQR) (25th and 75th percentile). Qualitative variables were expressed in terms of percentages and were analyzed using the chi-squared test (or Fisher Exact Test when necessary). The normal distribution of the variables was analyzed using the one sample Kolmogorov-Smirnov test. The association of quantitative variables (hs-Tg and rhTSH-Tg) was calculated using Pearson’s linear correlation coefficient. A Receiving Operating Characteristics (ROC) curves analysis was performed to determine the accuracy of rhTSH-Tg and hs-Tg at 12 months after completing initial treatment, as a marker of long-term DTC status defining disease-free staging as an Excellent Response (ER) at DRS in the last monitoring visit. ER was defined according to 2015 ATA Guidelines: no evidence of structural disease on imaging AND hs-Tg on levotiroxine <0.2 ng/ml OR stimulated Tg <1 ng/ml and no AbTg [2]. The area under the curve (AUC) and cut-off points for the highest sensitivity (Sens) and specificity (Sp) for both hs-Tg and rhTSH-Tg were analyzed. Finally, Sens, Sp and predictive negative (NPV) and positive value (PPV) were evaluated for each cut-off points considering the existence or lack of ER at DRS in the last follow-up visit. Finally, differences between DRS according to initial hs-Tg or rhTSH-Tg levels compared to DRS at last follow-up visit were also evaluated. The accepted level of statistical significance was 5% (*p* < 0.05). The SPSS statistical software package, version 17.0 (SPSS Inc., Chicago, IL, United States), was used for analysis.

## Results

In total, 114 patients were evaluated (77% female) with a mean age at diagnosis of 46.4 ± 14.1 years and a median and IQR follow-up of 6.7 [3.1–8.0] years since diagnosis. The mean tumor size was 1.8 ± 1.3 cm in diameter. The predominant histology found was papillary carcinoma (89.5%). The risk of recurrence at diagnosis were 61.4% and 14.0% for patients at low and high risk, respectively (Table 1).

**Table 1:** Basal characteristics of patients with differentiated thyroid carcinoma

Basal characteristics	Mean ± SD/Percentage
Number of patients	114
Female (%)	77.2
Age at cancer diagnosis (years)	46.4 ± 14.1
Percentage of patients older than 55 years old	36.0%
Years of evolution from the diagnosis to the last visit -median- [IQR]	6.7 [3.1–8.0]
Size of main carcinoma (cm)	1.8 ± 1.3
Average number of [131]I treatments	1.4 ± 0.6
Total cumulative [131]I dose (MBq)	5.661 ± 3.160
Type of thyroid cancer	%
Papillary	89.5
Classic	53.5
Follicular variant	28.9
Aggressive variants	7.1
Follicular	10.5
Oncocytic variant	58.1
Other variants	41.9
Histological baseline characteristics	%
Multifocal	50.5
Capsular invasion	51.9
Vascular invasion	13.1
Tumor staging	%
Stage I	82.4
Stage II	7.9
Stage III	8.8
Stage IV	0.9
Risk of recurrence	%
Low risk of recurrence	61.4
Medium risk of recurrence	24.6
High risk of recurrence	14.0

*SD* standard desviation, *IQR* interquartile range (percentile 25–percentile 75)

Table 2 shows baseline, intermediate (72 h after the first rhTSH injection) and rhTSH-stimulated (3 days after the second rhTSH injection) TSH, free T4, Tg and AbTg levels assessed 12 months after the completion of initial treatment (thyroidectomy and [131]I ablation).

**Table 2:** Baseline, intermediate (72 h after the first rhTSH injection) and rhTSH-stimulated (3 days after the second rhTSH injection) TSH, free T4, Thyroglobulin and Thyroglobulin antibodies levels assessed 12 months after completion of initial treatment (thyroidectomy and [131]I ablation)

	Baseline	Intermediate	rhTSH stimulated
TSH (mUI/L)	1.5 ± 1.2	151.4 ± 61.0	22.7 ± 16.4
Free T4 (ng/dl)	1.6 ± 0.3	1.6 ± 0.3	1.4 ± 0.6
Thyroglobulin (ng/ml)	0.4 ± 0.8	1.3 ± 3.0	2.1 ± 5.3
thyroglobulin antibodies (UI/ml)	11.0 ± 7.5	10.9 ± 7.4	11.6 ± 6.0

**Fig. 1:**
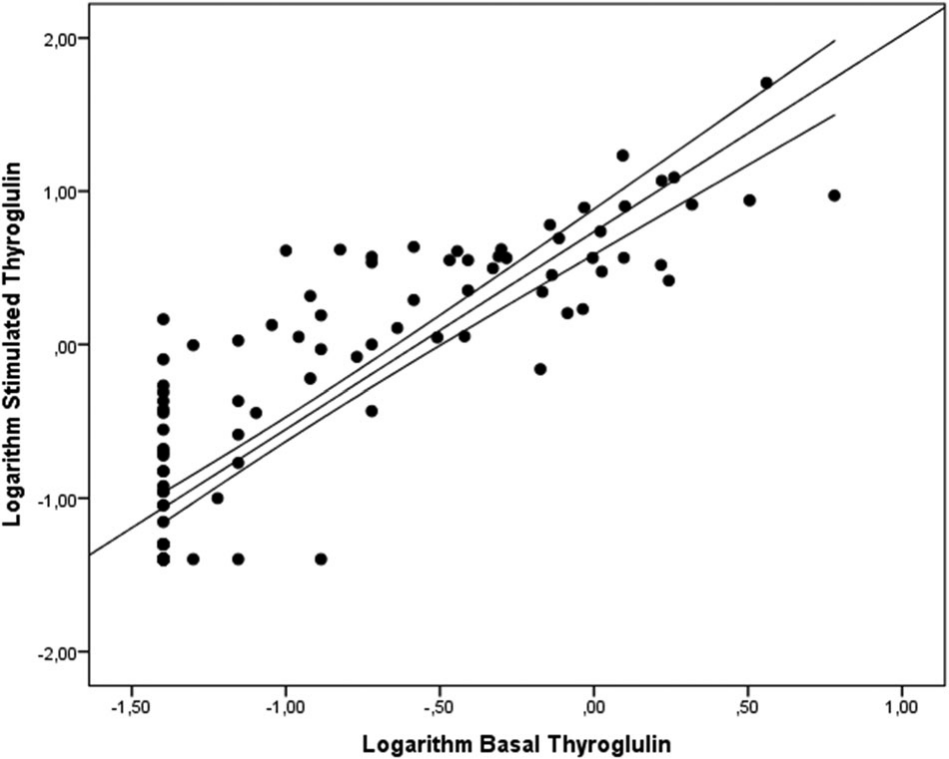
Correlation between stimulated rhTSH thyroglobulin and basal thyroglobulin levels on a logarithmic scale assessed 12 months after completion of initial treatment

When assessing the correlation between rhTSH-Tg and baseline hs-Tg at 12 months after the completion of initial treatment, a statistically significant strong positive linear correlation was found (*r* = 0.864, *p* < 0.001) (Fig. 1).

**Fig. 2:**
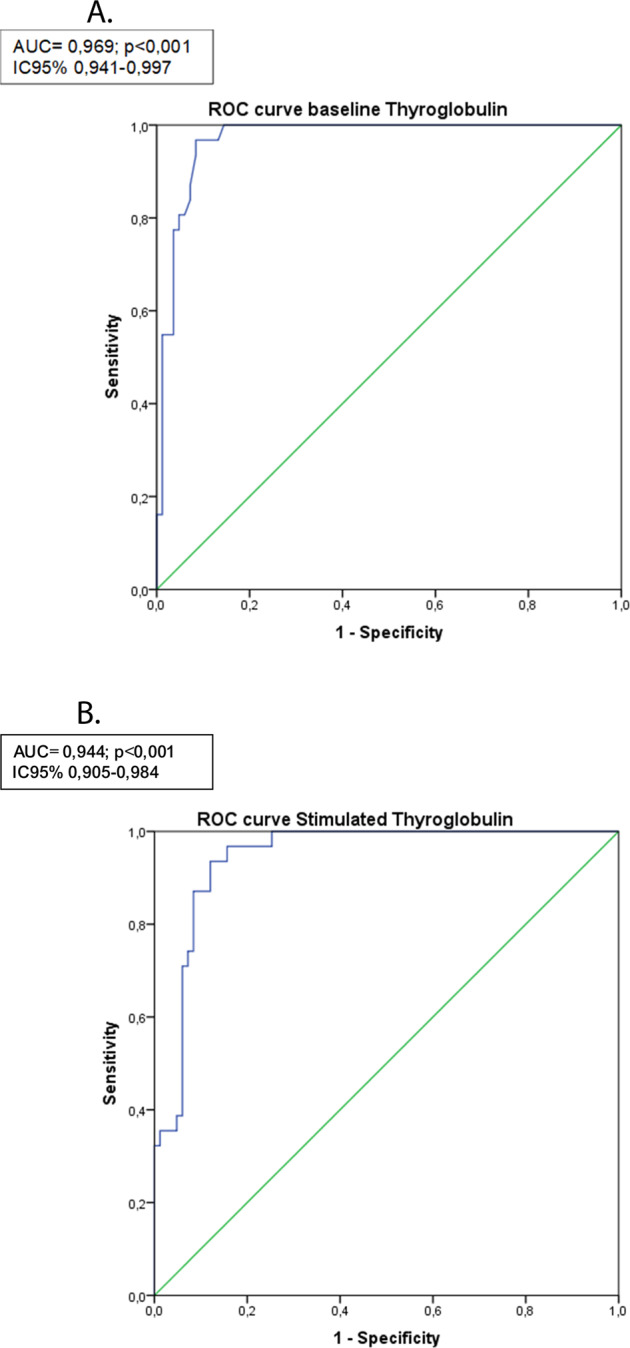
**A** ROC curve for basal thyroglobulin. **B** ROC curve for rhTSH stimulated thyroglobulin

ROC curve analysis was performed to evaluate the accuracy of rhTSH-Tg and baseline hs-Tg at 12 months after completing initial treatment as a marker of long-term DTC status. We analyzed the overall performance of the initial assessment of Tg levels with respect to DRS at the last follow-up visit, defining disease-free as an Excellent Response (ER) at the last monitoring visit. The AUC showed good diagnostic accuracy for both baseline hs-Tg (AUC = 0.969; *p* < 0.001; 95% CI 0.941–0.997) and rhTSH-Tg (AUC = 0.944; *p* < 0.001; 95% CI 0.905–0.984) (Fig. 2a, b). However, hs-Tg AUC was significantly better than rhTSH-Tg AUC.

The highest Sens and Sp hs-Tg and rhTSH-Tg cutoff points were 0.110 ng/dl and 0.815 ng/dl, respectively (Fig. 2a, b). According to the ROC cutoff points, the diagnostic predictive values (Sens, Sp, NPV and PPV) of initial hs-Tg and rhTSH-Tg levels were evaluated with respect to the existence of ER in the DRS at the last follow-up evaluation. Hs-Tg cutoff point showed S = 100%, Sp = 84.3%, NPV = 100% and PPV = 70.5%. At the same time, the rhTSH-Tg cutoff point showed a significantly worse diagnostic performance: S = 96.8%, Sp = 84.3%, PPV = 69.7% and NPV = 98.6%

Initial DRS according to hs-Tg and rhTSH-Tg levels was evaluated compared to DRS at the last follow-up visit.

With regard to the 83 patients with an ER at the last DTC assessment, 7.2% and 13.3% were incorrectly classified as biochemical incomplete response (BIR) or indeterminate response (IR) according to initial hs-Tg and rhTSH-Tg DRS, respectively (*p* < 0.05). On the other hand, patients with BIR at final DRS assessment were more frequently classified as IR at initial evaluation with rhTSH-Tg (80%) than with hs-Tg (40%) (*p* < 0.05). Finally, patients with IR at final DRS assessment were more frequently misclassified as ER at initial evaluation with hs-Tg (20%) than with rhTSH-Tg (6.7%) (*p* < 0.05) (Table 3a, b).

**Table 3:** **a** Initial hs-Tg dynamic risk stratification (DRS) compared to DRS at last follow-up visit. **b** Initial rhTSH-Tg dynamic risk stratification (DRS) compared to DRS at last follow-up visit

	Last follow-up visit dynamic risk stratification (DRS)				
	Excellent response	Biochemical incomplete response	Structural incomplete response	Indeterminate response	Total
Initial baseline hs-Tg					
Excellent response	77	1	0	3	81
Biochemical incomplete response	1	8	1	1	11
Indeterminate response	5	6	0	11	22
Total	83	15	1	15	114
Initial stimulated rhTSH-Tg					
Excellent response	72	1	0	1	74
Biochemical incomplete response	0	2	0	0	2
Indeterminate response	11	12	1	14	38
Total	83	15	1	15	114

*Hs-Tg* high sensitive thyroglobulin, *rhTSH-Tg* recombinant human TSH stimulated Thyroglobulin

## Discussion

Diagnostic, treatment and follow-up DTC guidelines have recently evolved towards a less aggressive approach that ensures adequate monitoring, especially in less aggressive forms [2]. In fact, the definition of ER in DRS is based exclusively on the absence of imaging findings (mainly cervical ultrasound) and hs-Tg levels on levothyroxine treatment <0.2 ng/mL or rhTSH-Tg < 1 ng/mL [2]. However, the absence of imaging findings but elevated Tg levels would classify these patients as IR (hs-Tg 0.2–1.0 ng/mL or rhTSH-Tg 1–10 ng/mL) or BIR (hs-Tg > 1.0 ng/mL or rhTSH-Tg > 10 ng/mL). Therefore, Tg is a key marker of recurrence or persistence disease in the long-term evolution of DTC [12]. Nevertheless, these DRS cut-off points for Tg are arbitrary, mainly based on retrospective studies and may be inconsistent when Tg is measured after stimulation (rhTSH-Tg) or under suppressive therapy (hs-Tg) for the same patient [8, 12].

DRS allows the reclassification of DTC status throughout its evolution independent of its initial recurrence risk. Those with structural incomplete response (SIR) have the highest risk of morbidity and mortality in DTC. Hence, the importance of Tg as a predictive marker of SIR [2].

During the last two decades, significant progress has been made in the measurement of hs-Tg. The hs-Tg assays have a tenfold higher sensitivity than first-generation assays, which could theoretically obviate the need for Tg stimulation (either by thyroid hormone withdrawal or rhTSH) [13]. However, the implications of DRS reclassification by rhTSH-Tg levels are unclear, especially in those patients with BIR and IR [14, 15].

In the present study, we evaluated both questions: (1) the long-term predictive value of hs-Tg and rhTSH-Tg 12 months after completion of initial treatment (total thyroidectomy and [131]I ablation) in a cohort with median follow-up of 6.7 years; and (2) the disagreements in DRS according to hs-Tg or rhTSH-Tg levels and their implications on the follow-up.

In the present article, the predictive value of ER at the last follow-up visit for Tg levels 12 months after completing initial treatment showed a high NPV. In fact, a hs-Tg cutoff point of 0.110 ng/mL showed an NPV of 100%. That is, all patients with an undetectable hs-Tg level under levothyroxine treatment 12 months after completing treatment with total thyroidectomy and [131]I ablation remained in ER throughout follow-up. These results agree with those previously published in which the NPV is estimated to be around 97–99%. Thus, the risk of a false negative at undetectable hs-Tg levels would be no more than 1% [8, 12].

Surprisingly, the results obtained for rhTSH-Tg did not improve the overall test performance with respect to disease status at the last follow-up visit. In fact, for a rhTSH-Tg cutoff point of 0.815 ng/dl the NPV and PPV of the test worsened slightly with respect to the predictive ability of hs-Tg. These results were due to a single 44-year-old female patient with follicular variant CDT and IR after a follow-up longer than 8 years (last follow-up visit hs-Tg = 0.22 ng/dl) and rhTSH-Tg from 0.19 to 0.37 ng/dl. Despite the borderline positivity in hs-Tg levels, it did not reach the cut-off point established by International Guidelines for rhTSH-Tg DRS, not even the one with the highest sensitivity/specificity calculated for the present study. Although it is true that the risk of developing structural disease in this patient is very low, it is undeniable that the cut-off points established for the definition of the DRS are arbitrary and may even cause divergences in DRS classification depending on whether rhTSH-Tg or hs-Tg is assessed, even though there is a clear correlation between both values (Fig. 1). In any case, our results support the inefficacy of rhTSH-Tg in predicting long-term response to DTC, making this costly procedure, which has a negative impact on patients´ quality of life, especially in those with an ER after completing initial treatment (certainly the most common subgroup in CDT), unnecessary [12, 16, 17].

At the same time, special attention should be paid to the lower PPV in any Tg evaluated. Tg levels 12 months after the completion of initial treatment may present false-positive results. In fact, 15.7% of patients identified as ER at the end of follow-up were classified as (false) positives in the initial assessment. The persistence of healthy thyroid tissue due to the temporal proximity of the [131]I ablative dose, the greater sensitivity of ultrasensitive tests and even the evolution towards spontaneous resolution in a high proportion of DTC could explain this fact [18]. In any case, our results are similar and even slightly superior to those previously described [12]. It supports the usefulness of hs-Tg as a discrimination tool for those patients with initial ER whose frequency and intensity of follow-up could be reduced, with the consequent cost savings, optimization of resources and reduction of patient anxiety during follow-up [19].

Finally, the DRS assess the risk of recurrence at any time during the DTC evolution in relation to the treatment response (ATA2015). In this sense, those patients without structural disease by imaging will define their DRS and therefore the risk of recurrence, exclusively based on Tg levels [4, 6]. These Tg cut-off points are arbitrary, based on retrospective studies and may differ depending on whether they are classified by hs-Tg or rhTSH-Tg [12]. In the present study, DRS classification using rhTSH-Tg 12 months after completing initial treatment overestimated the number of patients initially identified as IR in both ER and BIR groups at the last follow-up visit by more than two-fold. However, it provided a slightly better predictive value in those patients with an initial IR using rhTSH-Tg (93.3%) than hs-Tg (73.3%). Despite these apparent discrepancies between DRS classifications, the risk of recurrence associated with BIR or IR remains a subject of debate and depends on numerous variables (progressive increase in Tg levels, doubling time, etc.) and may evolve towards spontaneous resolution without further treatment in a large number of patients [18, 20]. Moreover, as shown in Table 3, 13.3% of patients with IR by rhTSH-Tg spontaneously progress to ER during follow-up. In fact, the clinical relevance of detectable but only slightly elevated hs-Tg levels is still unclear.

Although there has been a recent study aimed at evaluating the usefulness of rhTSH-Tg 5 years after the completion of initial treatment to reclassify DRS in those cases with IR or BIR [15], a regular hs-Tg monitoring strategy would probably have similar results with lower costs and less patient anxiety due to the overall good prognosis [21].

The present study has certain limitations to be considered. First, classic studies attribute to DTC the risk of recurrence even several decades after diagnosis [22], which would reduce the predictive value of hs-Tg after completing the initial treatment, even in a cohort with an average follow-up of almost 7 years. However, more recent studies have shown that more than 80% of recurrences are identified in the first 5 years [23], which would support the clinical relevance of our results. This increase in the early detection of recurrences is essentially due to the technical improvement on cervical ultrasound imaging and hs-Tg during the last decade. In this sense, the high NPV of undetectable hs-Tg and the absence findings on imaging evaluation, added to the higher risk of recurrence within the first 5 years, might support the possibility of extending follow-up and even definitively discharging those patients with low-risk DTC after this period [19]. Finally, the smaller number of patients with IR/BIR compared to those with ER could reduce the statistical significance in those patients; however, the prospective and longitudinal design of the present study as well as its similarity with other current cohorts ensure its representativeness and reflect the reality of DTC follow-up [24].

In conclusion, in those patients with ER 12 months after completing initial treatment (total thyroidectomy and [131]I ablative treatment), the rhTSH-Tg did not provide relevant information. An ER after initial treatment was associated with a high NPV. In those patients with IR or BIR, rhTSH-Tg identified a greater number of patients with IR, with no apparent practical implication during follow-up.

## Supplementary Information

Suplementary data
