# Supplementary material for: Predictive value and dynamic risk stratification of high sensitive basal or stimulated thyroglobulin assay in a long-term thyroid carcinoma cohort
Source: Endocrine. 2023 Feb 23;81(1):116–22. doi: 10.1007/s12020-023-03320-y (PMC10239375; doi:10.1007/s12020-023-03320-y)
Supplement: Supplementary file 1 — Suplementary data [file 12020_2023_3320_MOESM1_ESM.docx]

| **CHARACTERISTICS** | **EXCELLENT RESPONSE** | **NON-EXCELLENT RESPONSE** | **p** |  |
| --- | --- | --- | --- | --- |
| Number of patients | 83 | 31 | - |  |
| Female (%) | 77.1 | 77.4 | ns |  |
| Age at cancer Diagnosis (years) | 46.3±13.5 | 46.8±15.7 | ns |  |
| Percentage of patients older than 55 years old | 38.6 | 29.0 | ns |  |
| Years of evolution from the diagnosis to the last visit -median- [IQR] | 6.1 [3.3-7.0] | 6.9 [3.7-8.1] | ns |  |
| Size of main carcinoma (cm) | 1.7±1.1 | 2.1±1.6 | ns |  |
| Average number of [131]I treatments | 1.2±0.3 | 1.8±0.7 | <0.001 |  |
| Total cumulative [131]I dose (MBq) | 4,643±2,234 | 8,251±3,811 | <0.001 |  |
|  | |  |  |  |
| **TYPE OF THYROID CANCER** | **%** | | ns |  |
| Papillary | 88.0 | 93.5 |  |  |
| Follicular | 12.0 | 6.5 |  |  |
|  | | | |  |
| **HISTOLOGICAL BASELINE CHARACTERISTICS** | **%** | | |  |
| Multifocal | 50.6 | 48.4 | ns |  |
| Capsular invasion | 45.8 | 67.7 | <0.05 |  |
| Vascular invasion | 12.0 | 16.1 | ns |  |
|  | |  |  |  |
| **TUMOR STAGING** | **%** | | <0.05 |  |
| Stage I | 90.4 | 61.3 |  |  |
| Stage II | 4.8 | 16.1 |  |  |
| Stage III | 4.8 | 19.4 |  |  |
| Stage IV | 0.0 | 3.2 |  |  |
|  | |  |  |  |
| **RISK OF RECURRENCE** | **%** | | <0.01 |  |
| Low risk of recurrence | 67.5 | 45.2 |  |  |
| Medium risk of recurrence | 26.5 | 19.4 |  |  |
| High risk of recurrence | 6.0 | 35.5 |  |  |

SD: standard desviation

IQR: interquartile range (percentile 25 - percentile 75)

**Supplementary table 1**. Patients´ features with or without excellent response at final follow up visit
